# Transgenic Expression of *Cacna1f* Rescues Vision and Retinal Morphology in a Mouse Model of Congenital Stationary Night Blindness 2A (CSNB2A)

**DOI:** 10.1167/tvst.9.11.19

**Published:** 2020-10-14

**Authors:** Derek M. Waldner, Kenichi Ito, Li-Li Chen, Lisa Nguyen, Robert L. Chow, Amy Lee, Derrick E. Rancourt, Francois Tremblay, William K. Stell, N. Torben Bech-Hansen

**Affiliations:** 1 Graduate Department of Neuroscience, Cumming School of Medicine, University of Calgary, Calgary, AB, Canada; 2 Department of Biochemistry and Molecular Biology, University of Calgary, Calgary, AB, Canada; 3 Department of Biology, University of Victoria, Victoria, BC, Canada; 4 Department of Medical Genetics, Cumming School of Medicine, University of Calgary, Calgary, AB, Canada; 5 Department of Molecular Physiology and Biophysics, Department of Otolaryngology Head-Neck Surgery and Department of Neurology, University of Iowa, Iowa City, IA, USA; 6 Department of Ophthalmology and Visual Sciences, Faculty of Medicine, and Clinical Vision Sciences Program, Faculty of Health Dalhousie University, NS, Canada; 7 Department of Cell Biology and Anatomy and Department of Surgery, Hotchkiss Brain Institute, and Alberta Children's Hospital Research Institute, Cumming School of Medicine, University of Calgary, Calgary, AB, Canada; 8 Department of Medical Genetics, and Department of Surgery, Alberta Children's Hospital Research Institute, and Hotchkiss Brain Institute, Cumming School of Medicine, University of Calgary, Calgary, AB, Canada

**Keywords:** CSNB2A, Cav1.4, Cacna1f, channelopathies, calcium channels

## Abstract

**Purpose:**

Congenital stationary night blindness 2A (CSNB2A) is a genetic retinal disorder characterized by poor visual acuity, nystagmus, strabismus, and other signs of retinal dysfunction resulting from mutations in *Cacna1f—*the gene coding for the pore-forming subunit of the calcium channel Ca_V_1.4. Mouse models of CSNB2A have shown that mutations causing the disease deleteriously affect photoreceptors and their synapses with second-order neurons. This study was undertaken to evaluate whether transgenic expression of *Cacna1f* could rescue morphology and visual function in a *Cacna1f*-KO model of CSNB2A.

**Methods:**

Strategic creation, breeding and use of transgenic mouse lines allowed for Cre-driven retina-specific expression of *Cacna1f* in a CSNB2A model. Transgene expression and retinal morphology were investigated with immunohistochemistry in retinal wholemounts or cross-sections. Visual function was assessed by optokinetic response (OKR) analysis and electroretinography (ERG).

**Results:**

Mosaic, prenatal expression of *Cacna1f* in the otherwise *Cacna1f*-KO retina was sufficient to rescue some visual function. Immunohistochemical analyses demonstrated wild-type-like photoreceptor and synaptic morphology in sections with transgenic expression of *Cacna1f*.

**Conclusions:**

This report describes a novel system for Cre-inducible expression of *Cacna1f* in a *Cacna1f*-KO mouse model of CSNB2A and provides preclinical evidence for the potential use of gene therapy in the treatment of CSNB2A.

**Translational Relevance:**

These data have relevance in the treatment of CSNB2A and in understanding how photoreceptor integration might be achieved in retinas in which photoreceptors have been lost, such as retinitis pigmentosa, age-related macular degeneration, and other degenerative conditions.

## Introduction

The family of retinal disorders called congenital stationary night blindness is a subset of inherited retinal conditions that are generally non-progressive, global and predominantly associated with dysfunction of the rod system. Genetic mutations causing congenital stationary night blindness (CSNB) occur in genes associated with phototransduction, retinoid recycling, presynaptic glutamate release, or postsynaptic glutamate-induced signaling within the retina. To date, 17 genes have been identified, with over 360 distinct mutations, causing myriad clinical manifestations.^1^^,^^2^ CSNB-causing abnormalities in the fundus (caused by disruption of retinoid recycling) can be distinguished from those with a normal fundus, which are further classified into Riggs-type and Schubert-Bornschein–type. Riggs-type CSNB is caused by a disruption of phototransduction within photoreceptors, whereas Schubert-Bornschein–type is caused by dysfunctions in signaling from photoreceptors to bipolar cells.^3^^,^ ^4^ Schubert-Bornschein–type CSNB is further divided into Type 1 (formerly known as “Complete”) and Type 2 (formerly known as “Incomplete) CSNB, on the basis of electroretinographic characteristics.^1^^,^^5^^–^^8^

Type 2 CSNB with an X-linked pattern of inheritance (CSNB2A) is the single most common form of CSNB, attributable to mutations in a single gene—*CACNA1F.*^1^^,^^9^^–^^11^ (CSNB2A has also been referred to as Åland eye disease, incomplete CSNB, and X-linked cone-rod dystrophy 3. Alternative nomenclatures have also been proposed including “congenital rod-cone synaptic dysfunction” and “post-transduction defect.” For simplicity, CSNB2A will be used throughout this article.^1^^,^^11^) *CACNA1F* codes for the pore-forming α_1F_ subunit of the L-type voltage-gated calcium channel (VGCC): Ca_V_1.4. Ca_V_1.4 is the voltage-gated calcium channel located at photoreceptor terminals, where it mediates the inward calcium current necessary for glutamate release. Thus, in its absence or dysfunction caused by genetic mutation, synaptic transmission from both rods and cones is impaired or absent (For a review of the physiological functions of Ca_V_1.4, see Waldner et al.^12^). This deficit in synaptic transmission is reflected in electroretinogram (ERGs), which show abnormalities in both scotopic and photopic responses. ERG a-waves are largely preserved (indicating functional phototransduction in photoreceptors), whereas b-waves have markedly reduced amplitude, resulting in an overall electronegative waveform.^7^^,^^13^ Symptoms associated with CSNB2A include poor visual acuity, nystagmus, strabismus, refractive errors, and color vision defects, with marked phenotypic variability between individuals.^14^^–^^16^

Several CSNB2A mouse models with loss-of-function *Cacna1f* mutations have now been established and extensively characterized.^17^^–^^20^ These data have shown that in the absence of α_1F_/Ca_V_1.4, synaptic ribbons of photoreceptor presynaptic membrane do not anchor to the presynaptic membrane or adopt their mature, elongated morphology.^19^^,^^21^^,^^22^ For this and other reason(s), it has been suggested that Ca_v_1.4 might serve as a scaffold for presynaptic proteins that are necessary for synaptogenesis.^12^^,^^19^^,^^23^^,^^24^ This disruption of presynaptic elements leads to failure of synaptogenesis between photoreceptors and its typical synaptic partners—bipolar and horizontal cells—which subsequently exhibit dendritic sprouting into the outer nuclear layer with time.^18^^,^^20^^,^^22^^,^^25^ Photoreceptors, by contrast, exhibit substantial axonal abnormalities and degenerate with time.^17^^,^^18^^,^^21^ The cumulative effect of these morphologic changes is to render these mice completely blind by all behavioral and electrophysiological assessments performed to date.^18^^,^^26^^,^^27^ This discrepancy between visual function in *Cacna1f*-KO mice and their analogous human counterparts is not yet fully understood.

These mouse models and the established data on the molecular basis of retinal dysfunction now provide opportunities to evaluate the potential for preventing or reversing the mutation effects via gene therapy. Most recently, Laird et al. used in vivo electroporation to express Ca_V_1.4 transiently in a *Cacna1f*-KO model and found some evidence of mature synaptogenesis and possible improvement in visual function based on performance in visually-guided tasks. However, this experiment was limited by efficacy of electroporation, with only ∼100 synaptic terminals expressing the transgene per retinal section.^28^ The current article reports efforts to investigate this experimental question in another way, by strategic creation and use of transgenic mouse lines to express *Cacna1f* conditionally. This can be achieved by use of the Z/EG (LacZ/eGFP) system, with which coexpression of *Cre* recombinase can facilitate expression of desired genes in an observable manner with the dual reporters indicating regions of expression and recombination.^29^ Different *Cre* transgenes can be used to control temporal and spatial expression of genetic elements included in the modified Z/EG transgene, allowing for multiple uses of single transgenic Z/EG lines. In this work, a transgenic Z/EG line including *Cacna1f (iZEG:Cacna1f)* was validated by *Cre* recombination under control of the Pax6 α-enhancer/PO promoter (*Pax6::Cre),* which has early (prenatal) *Cre* expression, largely restricted to progenitor cells in the peripheral retina.^30^ Subsequent breeding with the *Cacna1f^G305X^* mouse was performed to verify whether the recombined *iZEG:Cacna1f* transgene could restore formation of synaptic ribbons and visual function, as assessed by immunohistochemical, optokinetic response, and electroretinographic analysis.

These data are relevant, not only for the potential of developing CSNB2A therapies, but also for the promising technique of photoreceptor transplantation to treat retinal degeneration. As the primary deficit observed in the *Cacna1f*-KO retina is a lack of synaptogenesis with minimal early degeneration or inner retinal changes, restoration of α_1F_ expression should answer whether it is possible for photoreceptors to integrate into existing retinal circuitry after the normal developmental period—a problem shared by therapeutic efforts in transplantation of rods and cones for treatment of degenerative retinal diseases.^12^^,^^17^^,^^21^ The data in this report verify the utility of this system for rescuing visual function and retinal cell morphology in *Cacna1f*-KO mice and provide the foundational studies for future research using this approach.

## Materials and Methods

### Animal Care and Ethics

Mice were maintained in the Mouse Double Barrier Unit of the Clara Christie Centre for Mouse Genomics under a 12:12-hour light/dark cycle. All mouse experiments were approved by the University of Calgary Animal Care Committee, under protocol numbers AC12-0090 and AC16-0119, or Dalhousie University Committee on Laboratory Animals (19-058), and they adhered to the animal care guidelines established by the Canadian Council on Animal Care and the ARVO Statement for the Use of Animals in Ophthalmic and Vision Research. *Pax6 α-enhancer/PO Promoter::Cre-IRES-GFP* mice (henceforth referred to as *Pax6::Cre*) were a kind gift of Dr. Carol Schuurmans.^30^^,^^31^ *Cacna1f*^G305X^ mice were used as the CSNB2A/*Cacna1f*-KO model.^20^

### Design and Validation of the *iZEG:Cacna1f* Transgene Vector

The *iZEG:Cacna1f* transgene was designed and created as a double-reporter system at the University of Calgary Centre for Genome Engineering, following the work of Novak et al.^29^ Mouse *Cacna1f* cDNA in a gateway vector was ordered from GeneCopoeia (Rockville, MD, USA; no. MOC23581) and cloned into the multiple cloning site of the iZEG vector (http://www.cancerbiology.org/dnaizegmap.html), upstream of the eGFP reporter, and downstream of the constitutively expressed *LoxP*-flanked *LacZ* gene driven by the strong, universal, CAG promoter using recombineering technology.^32^ Additionally, an *EM7-BleoR* cassette was introduced downstream of the *eGFP* ORF via recombineering technology, to introduce a second *ScaI* restriction enzyme site for vector linearization The *iZEG:Cacna1f* vector was validated by DNA sequencing and by both in vitro and in vivo expression assays. For in vitro validation, HEK293T cells were cotransfected with the *iZEG:Cacna1f* vector, a *CMV::Cre* vector, and a *CMV* driven, nucleus-targeted red fluorescent protein (RFP) reporter control plasmid (*CMV::RFP)*, using the FuGENE HD Transfection Reagent according to the manufacturer's instructions (https://www.promega.ca/resources/protocols/technical-manuals/101/fugene-hd-transfection-reagent-protocol). In vivo validation was performed via electroporation of wildtype mouse retinae at P0.^33^ These retinas were imaged at P14.

### Generation of Transgenic Mice

The *iZEG:Cacna1f* transgenic mice were generated in the University of Calgary Centre for Genome Engineering, by pronuclear injection of *ScaI*-linearized *iZEG:Cacna1f* vector DNA into fertilized eggs of C57Bl/6J mice.^34^ Embryos at the two-cell stage were then transferred into the oviducts of pseudopregnant female mice and gestated. The resulting 84 offspring were evaluated for transgene integration by PCR genotyping of both tail snips and ear punches. This yielded six transgene-positive founder lines, each of which showed transmission of the *iZEG:Cacna1f* transgene to subsequent generations confirming integration into the germ line.

### Breeding Strategy and Genotyping

Producing mice of the various genotypes that were necessary for the aims of this research required multiple generations of sequential breeding. First, confirmed *iZEG:Cacna1f*^+^ mice were bred with *Pax6::Cre*^+^ mice to generate the *iZEG:Cacnalf^+^;Pax6::Cre^+^* mice. (Due to the complexity of transgenes in this article, steps have been taken to simply things for the reader. (1) Mice with *Pax6::Cre* and *iZEG:Cacna1f* transgenes are always hemizygous (+/0) for these genetic elements. A superscript + will be used to designate mice with these transgenes. (2) *Cacna1f*, and the corresponding gene *CACNA1F* in man, are located on the X-chromosome. Homozygous *Cacna1f*-KO females and hemizygous *Cacna1f*-KO males are collectively referred to as *Cacna1f^G305X^* or *Cacna1f*-KO.) Next, *iZEG:Cacnalf^+^;Pax6::Cre^+^* males were bred with homozygous *Cacna1f^G305X^* females to generate *iZEG:Cacnalf^+^;Pax6::Cre^+^;Cacna1f*^G305X^ males*,* which were the focus of the present study. Other mice resulting from these pairings were used as experimental controls or in subsequent breeding pairs, as appropriate. It should be noted that breeding pairs involving *iZEG:Cacna1f*^+^ mice tended to produce fewer pups than expected, with increased frequency of embryonic resorption compared with that in wild-type breeding pairs.

### Immunohistochemical Labeling

Whole-mounted retinas or retinal sections were obtained and prepared and immuno-labeled with appropriate antibodies as previously described.^17^ Antibodies used in this study are listed in the Table. For X-Gal staining, embryos were fixed overnight in 4% paraformaldehyde (PFA) solution, before equilibration in 30% (w/v) sucrose solution with 2 mmol/L MgCl_2_. Equilibrated embryos were then washed at room temperature 3 × 30 minutes in detergent solution (0.01% sodium deoxycholate, 0.02% NP-40, 2 mmol/L MgCl_2_ in phosphate-buffered saline solution [PBS]), followed by overnight incubation in staining solution (detergent solution + 5 mmol/L K_4_Fe(CN)_6_ 3 H_2_O + 5 mmol/L K_3_Fe(CN)_6_ + 0.8 mg/mL X-gal). Embryos were postwashed 3 × 5 minutes in ice-cold PBS solution and postfixed overnight in 4% PFA for subsequent observation or cryoprotection and sectioning. Imaging was performed as previously described, using Olympus FV1000 and Nikon A1R microscopy systems.^17^

**Table.** Antibodies Used for the Characterization of Transgenic Mouse Retinas

Antibody	Host Species	Form	Dilution	Source	Antigen	References
GFP (A-11222)	Rabbit	Affinity purified polyclonal antisera	1:1000	Thermo Fisher Scientific, Waltham, MA, USA	GFP isolated from *Aequorea victoria*	^35^
Beta-Galactosidase (40-1a)	Mouse	Purified mouse monoclonal IgG1	1:1000	Developmental Studies Hybridoma Bank, University of Iowa, Iowa CIty, IA, USA	Unknown	^36^
α1F	Rabbit	Affinity purified polyclonal antisera	1:5000	A. Lee, University of Iowa, Iowa City, IA, USA	Mouse α1F oligopeptide (1-20)	^19^
RIBEYE (Clone 16/CtBP2)	Mouse	Purified mouse monoclonal IgG1	1:1000	BD Biosciences, Mississauga, ON, Canada	Mouse RIBEYE oligopeptide (361-445)	^37^
Cone Arrestin	Rabbit	Affinity purified polyclonal antisera	1:1000	C. Craft, University of Southern California, Los Angeles, CA, USA	bovine CRX oligopeptide (279-292)	^38^
PKCα	Goat	Purified goat polyclonal IgG	1:1000	R&D Systems, Minneapolis, MN, USA	Recombinant human PKCα (604-672)	^39^

### Electrophysiological Studies

Electrophysiological investigations were carried out as previously described.^20^ Briefly, electroretinograms (ERGs) were recorded after overnight dark-adaptation, followed by ketamine HCl (100 mg/kg, intraperitoneally [i.p.]) and xylazine (10 mg/kg, i.p.) anesthesia, mydriasis with cyclopentolate HCl (0.5%; Alcon, Fort Worth, TX, USA) and corneas anesthesia with proparacaine HCl (0.5%; Alcon). At the end of experiments, animals were sacrificed with an overdose of pentobarbital (100 mg/kg, i.p.), and tail snips were collected.

Flash stimuli with maximum intensity of 10.1 log cd ∙ s ∙ m^−2^ and attenuated by interposed neutral density filters were presented in a Ganzfeld sphere (LKC Technologies, Gaithersburg, MD, USA). Silver-impregnated nylon fibers (Retina Technologies, Scranton, PA, USA) were laid down on both corneas, as active electrodes, while platinum electrodes (F-E2, Astro-Med Inc., Brossard, QC, Canada) were inserted subdermally on the nose as reference, and in the right hind leg as ground. The signal was amplified 10,000-fold, with an open bandwidth of 3 to 1000 Hz (P511; Grass Instruments, West Warwick, RI, USA) and was digitized (sampling rate of 1000 Hz, PCI 6281; National Instruments, Austin, TX, USA). Dark-adapted and photopic activities were collected.

The a-wave amplitude was measured from baseline to the most negative trough and the b-wave from the a-wave trough to the next maximum positive peak, after the signal was processed through a low-pass finite impulse response filter with a high cutoff frequency of 50 Hz using Matlab (MathWorks, Natick, MA, USA) to eliminate the interference of oscillatory potentials. In the absence of a b-wave, the peak amplitude at implicit time corresponding to average b-wave peak time in control (59ms) was used. Oscillatory potentials were isolated with a Butterworth filtering function of order 4, with a low cutoff frequency of 80 Hz.

After ERG recordings, the brain surface was exposed through craniotomy centered at 1.5 mm lateral on each side of the midline, 3 mm posterior to the bregma. Stereotaxic reference for the visual cortex was −2.5 mm anteroposterior axis, +2.0 mm mediolateral axis while the superior colliculi was reached at −4.0 mm anteroposterior axis, +1.0 mm mediolateral axis, and −1.5 mm dorsoventral axis.^40^ For visually evoked cortical potentials (VECPs), an insulated platinum electrode was lowered to the pial surface using a reference electrode inserted in the cervical muscles, away from retinal activity sources. One hundred responses to unattenuated flash stimuli were averaged, and four to six of those averaged potentials were collected. The response amplitude was measured from trough (implicit time ∼100 ms) to peak (∼150 ms). Multiunit activity was recorded after multiple single-flash recordings through an epoxylite-insulated high impedance tungsten microelectrode (Frederick Haer & Co, Bowdoinham, ME, USA), lowered to the superior colliculus stereotaxic position^40^ via a micromanipulator.

As proof of principle, recordings were performed in only two treated mice; consequently, results are simply contrasted individually to either control or *iZEG:Cacna1f^+^;Pax6::Cre^+^;Cacna1f^G305X^* mice, with these two groups being summarized using mean and 99% confidence interval (CI) and contrasted using nonparametric Wilcoxon signed-ranked test^41^ at a level of significance of 0.05. ERG responses from the two eyes were averaged to yield one value per animal; the same was performed for the VECPs.

### Optokinetic Response Analysis

The optokinetic response (OKR) was analyzed as previously described.^17^^,^^42^ In brief, the OptoMotry system was used to generate horizontally-drifting vertical sine-wave gratings, of various spatial frequencies, at a constant drift velocity of 12°/second. Spatial contrast threshold at a given spatial frequency was determined by a modified staircase procedure, in which the spatial luminance contrast was reduced stepwise until an optokinetic reflex could no longer be elicited. Percent contrast at threshold was converted to contrast sensitivity (CS) by the formula, CS =100/threshold % contrast. *iZEG:Cacna1f^+^;Pax6::Cre^+^;Cacna1f^G305X^* and *iZEG:Cacna1f^+^;Pax6::Cre^+^* mice were analyzed at about two to three months of age.

## Results

### Validation of the *iZEG:Cacna1f* Vector

**Figure 1.**
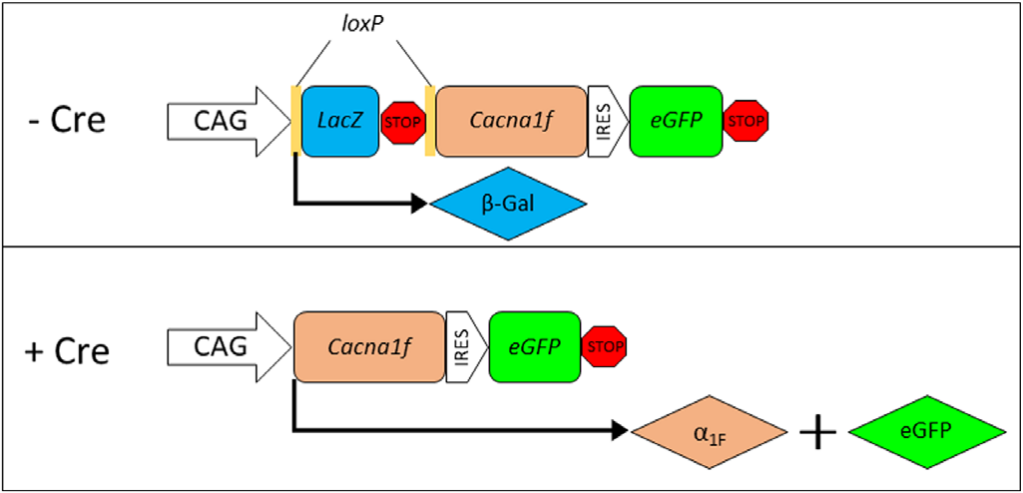
Schematic illustration of *iZEG:Cacna1f* transgene function. In the absence of *Cre*-mediated recombination, *LacZ* is expressed under control of the CAG promoter. With *Cre* expression, the *LacZ*-neo cassette is excised, resulting in expression of *Cacna1f* and *eGFP*.

The *iZEG:Cacna1f* vector was designed as a double-reporter system, in which independent protein markers can be visualized to identify cells expressing the transgene in which Cre-mediated recombination has (eGFP reporter), or has not (beta-galactosidase [β-Gal] reporter), occurred. *Cacna1f* will be coexpressed with *eGFP* only in cells in which Cre-mediated recombination has occurred (Fig. 1).

Proper function of the *iZEG:Cacna1f* vector was experimentally validated using in vitro and in vivo assays. HEK293 cells transfected with *CMV*::*Cre* driver, *iZEG:Cacna1f,* and RFP nuclear reporter plasmids synthesized eGFP protein, whereas cells transfected only with the latter two vectors did not. Further validation was performed in vivo by electroporation of *iZEG:Cacna1f* and the rod-specific *Nrl::Cre* vector into the developing murine retina at P0. Once again, eGFP synthesis in cells expressing both plasmids indicated that the *iZEG:Cacna1f* vector performed as designed (Supplementary Fig. S1).

### Characterization of *iZEG:Cacna1f* Transgene Expression

**Figure 2.**
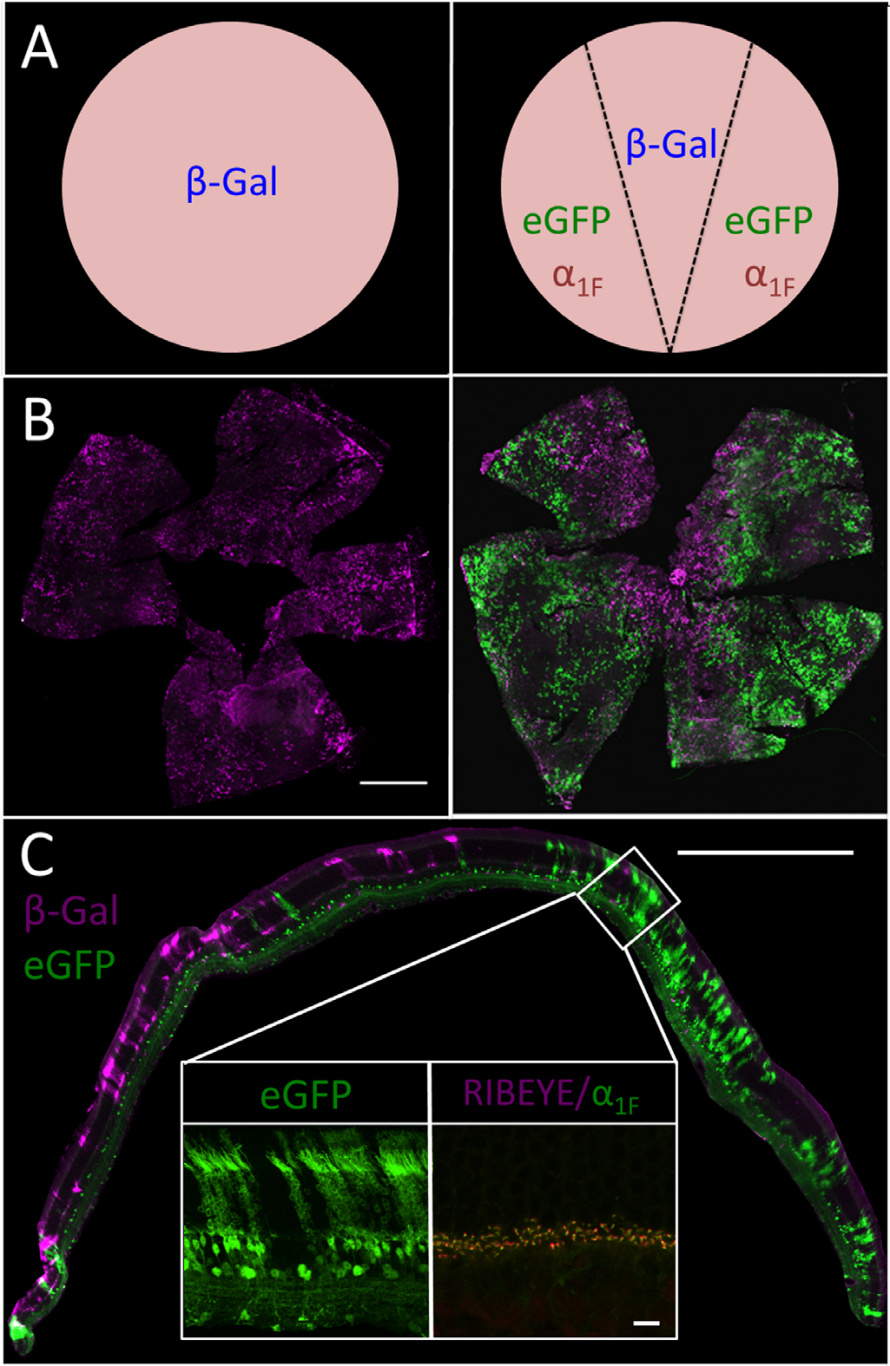
(A) Expected transgene expression in the *iZEG:Cacna1f*^+^ retina without (A) and with (B) co-expression of the *Pax6::Cre* driver transgene based on data from Cantrup et al. 2012.^31^ (B) Whole-mounted *iZEG:Cacna1f*^+^ (*Left*) and *iZEG:Cacna1f*^+^;*Pax6:*:*Cre*^+^ (*Right*) retinas, immunolabeled for beta-galactosidase (β-Gal; *magenta*) and GFP (*green*). *Scale bar*: 1000 µm. (C) Cross-section of an *iZEG:Cacna1f^+^;Pax6::Cre^+^* retina, immunolabeled for β-Gal and eGFP protein expression. *Insets* show a higher-resolution view of eGFP expression in a recombined, *iZEG:Cacna1f-*expressing region (*Left*), and ribbon morphology in that region by α_1F_ (*green*) and RIBEYE (*magenta*) immunolabeling. *Scale bars*: 500 µm (full image), 25 µm (*insets*).

Of the six founder lines originally shown to exhibit germ-line transmission of the *iZEG:Cacna1f* transgene, only one was found to express the recombination-negative reporter beta-galactosidase (β-Gal) as detected by X-gal staining of >50 E14.5 embryos. These *iZEG:Cacna1f*^+^ embryos exhibited widespread, albeit mosaic, labeling. Similar mosaic expression has been reported previously in transgenic predecessors of the iZEG vector system, with less than 5% of transgene-positive ES cell clones exhibiting strong, global β-Gal expression.^43^ This variable mosaicism has been attributed to random integration at chromosomal loci that are more or less prone to epigenetic silencing.^44^ Immunolabeling for β-Gal in retinal sections confirmed this mosaic expression pattern of the *iZEG:Cacna1f* transgene in the retina—with ∼10% to 20% of the area of any individual wholemount labeling for β-Gal, and expression primarily in photoreceptor cells (Fig. 2). Because wild-type *Cacna1f* expression within the mouse retina is limited at least primarily to photoreceptors, this restricted expression may be advantageous, because it would avoid the dysregulation of calcium currents caused by ectopic expression of α_1F_/Ca_V_1.4 in other retinal cells, after recombination.^12^

### Constitutive, Early In Vivo Recombination of *iZEG:Cacna1f* by *Pax6:*:*Cre*

In vivo recombination of the *iZEG:Cacna1f* transgene, driven by an endogenous *Cre* transgene driver, was then characterized. For this purpose we used *Pax6 α-enhancer/PO Promoter*::*Cre-IRES-GFP* (*Pax6:*:*Cre)* transgenic mice, which have a pre-established, early, constitutively retina-specific expression pattern confined to the retinal periphery.^30^^,^^31^ In these mice, one would expect *iZEG:Cacna1f*^+^_;_*Pax6::Cre^+^* retinas to exhibit labeling for β-Gal in photoreceptors of the central, non-recombined region, and for eGFP and α_1F_ in the peripheral regions, in which *Cre* has been expressed in retinal progenitor cells during development (Fig. 2A). Additionally, as the *Pax6::Cre* transgene contains an *IRES-GFP* cassette, GFP immunolabeling is expected in all cells that continue to express *Pax6* beyond retinogenesis—exclusively a subset of amacrine and ganglion cells, in the mouse.^45^

To detect *Pax6:*:*Cre*-mediated recombination, isolated retinas from *iZEG:Cacna1f^+^;Pax6:*:*Cre*^+^ and *iZEG:Cacna1f^+^* mice were co-immunolabeled for both reporters (β-Gal and eGFP) and then whole-mounted for visualization (Fig. 2B). The *iZEG:Cacna1f^+^;Pax6:*:*Cre*^+^ retinas exhibited the expected labeling pattern of both reporters, with recombination leading to eGFP expression in the periphery and β-Gal expressed in the central retina. The *iZEG:Cacna1f^+^* controls (lacking *Pax6::Cre-*driven recombination) exhibited global mosaic β-Gal immunoreactivity, but no eGFP, as expected. Cross-sections of *iZEG:Cacna1f^+^;Pax6:*:*Cre*^+^ retinas were similarly labeled for both reporters and also showed the expected retinal distribution (Fig. 2C). Interestingly, whereas nonrecombined regions showed β-Gal immunoreactivity almost exclusively in photoreceptor cells, eGFP expression as seen in cross-sections was *not* restricted to photoreceptors and *Pax6*-expressing amacrine/ganglion cells. The eGFP labeling was also observed in bipolar cells and some Pax6-negative amacrine cells of the INL—suggesting either that early recombination increases transgene expression, or that eGFP is detected more sensitively by immunohistochemistry (Fig. 2C, Left Inset). α_1F_ and RIBEYE immunolabeling of these sections showed no differences in ribbon morphology between recombined and nonrecombined regions (data not shown), indicating that transgenic α_1F_ does not disrupt the structural integrity of photoreceptor synapses. (Fig. 2C, right inset).

### Early Expression of α_1F_ Rescues Visual Function and Morphology in the *Cacna1f*-KO Retina

#### Behavioral Visual Analysis

**Figure 3.**
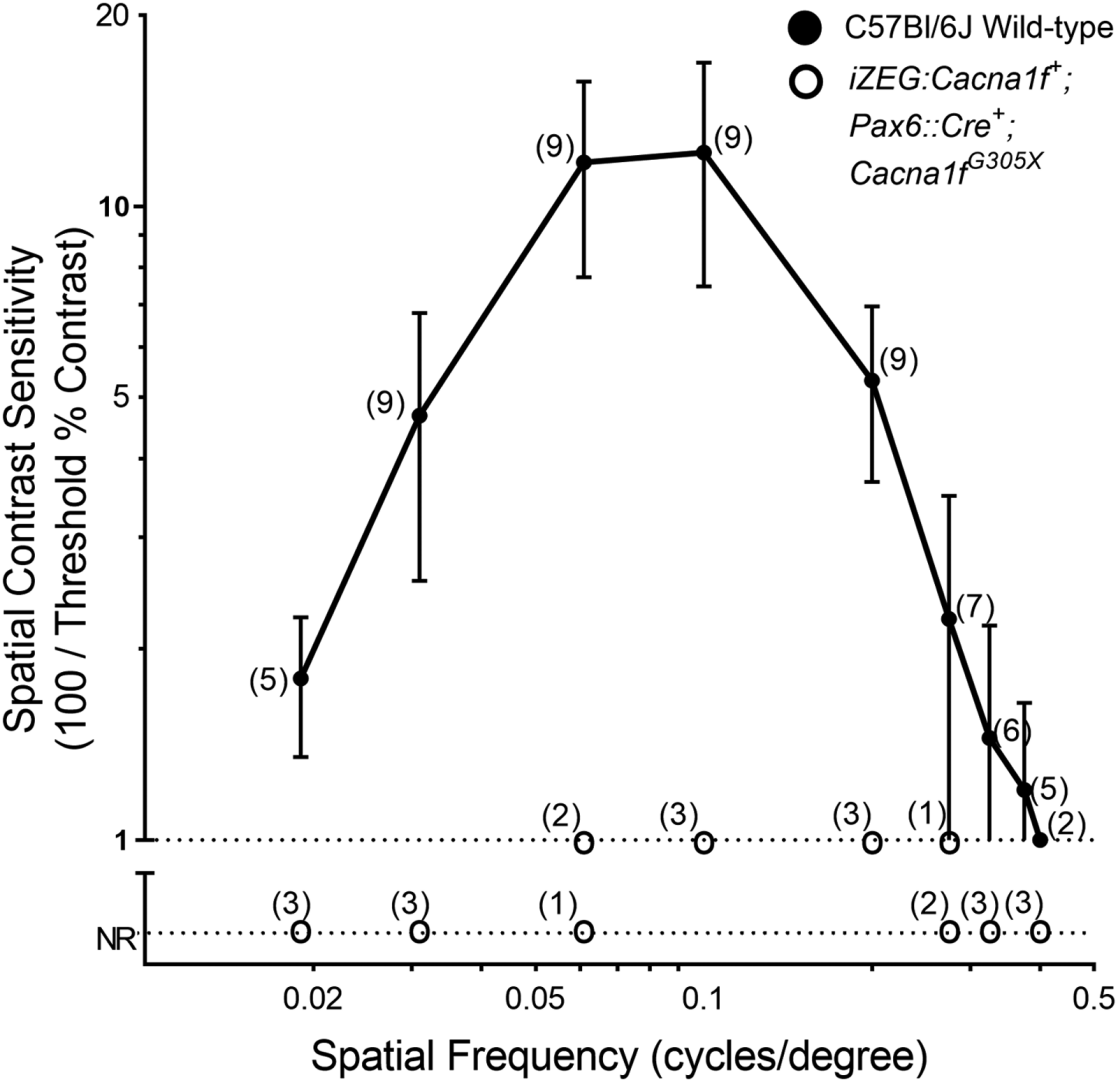
Comparison of spatial contrast sensitivities of *iZEG:Cacna1f^+^;Pax6::Cre^+^;Cacna1f^G305X^* mice (n=3) with those of C57Bl/6 wild-type (WT) mice (n = 9), using the optokinetic response (OKR). Wild-type data transposed from Waldner et al.^17^ *Error bars* clipped at axis limits.

After validating the expected expression of the *iZEG:Cacna1f* transgene with *Pax6:Cre* recombination, *iZEG:Cacna1f^+^;Pax6::Cre^+^;Cacna1f^G305X^* mice were analyzed to determine whether overexpression of *Cacna1f* cDNA could rescue visual function and normal synaptic morphology, with early, constitutive expression in the peripheral retina in the *Cacna1f*-KO background. OKR analyses of these mice showed exceedingly poor, but measurable, vision—limited to the highest contrast at the spatial frequencies that are optimal for mice (Fig. 3).

#### Electrophysiological Analysis

**Figure 4.**
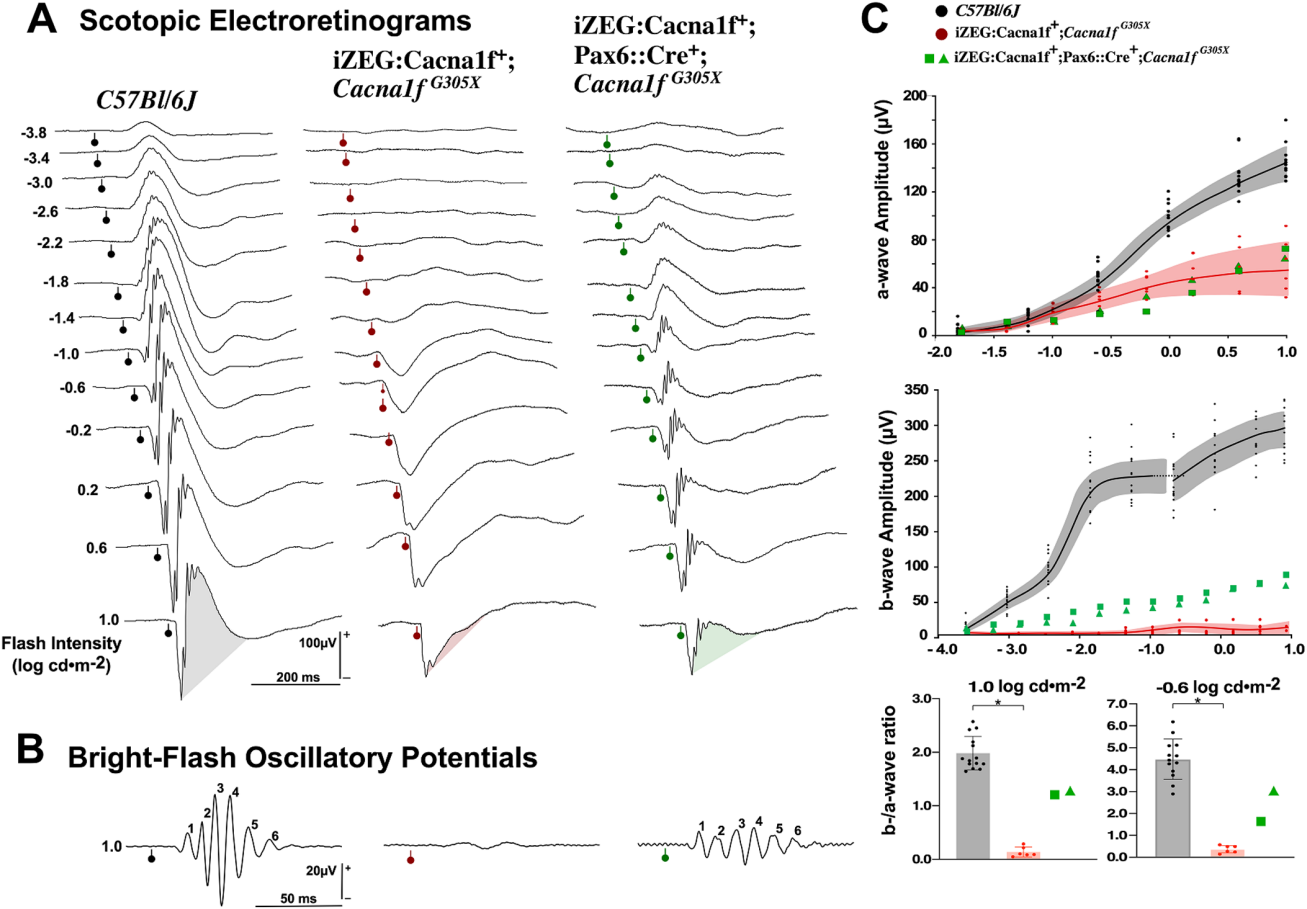
**(**A) Representative scotopic electroretinograms from a flash-intensity series for a C57Bl/6J control group (*left*), *Cacna1f* ^G305X^ KO mice (*center*), and *iZEG:Cacna1f^+^;Pax6::Cre^+^;Cacna1f^G305X^* mice (*right*). Flash intensities are in log cd ∙s ∙ m^−2^, and vertical bars with circles indicate flash onset time. *Shaded areas* help identify the preponderance of the b-wave component. (B) Oscillatory potentials isolated from the responses to highest intensity of the series. Calibration as indicated. (C) Intensity-response curves (mean and 99% CI (*shaded areas*)) for a-waves (*top*) and b-waves (*middle*), for the two groups along with individual data (*squares* and *triangles*) for the two treated animals. The a-/b-wave ratio computed from the brightest (1.0 log cd ∙ s ∙ m^−2^) and a lower (−0.6 0 log cd ∙ s ∙ m^−2^) flash response (mean ± SD, with individual data points) for the two groups, with individual data presented as *green squares* and *green triangles*. The control and *Cacna1f* ^G305X^ KO groups are significantly different (*Wilcoxon signed-ranked test), and the data from two treated individuals have no overlap with either groups.

**Figure 5.**
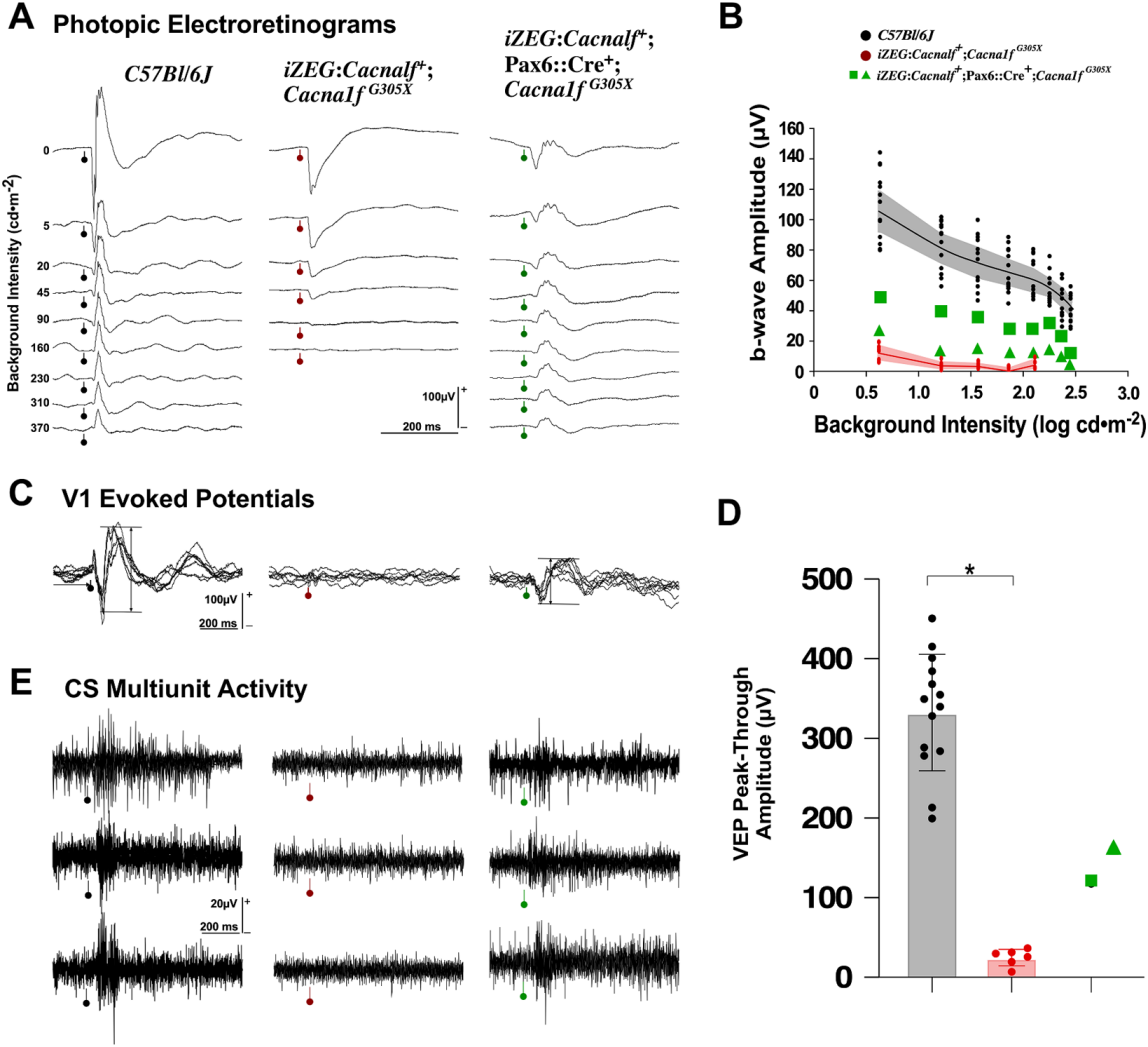
**(**A) Representative photopic electroretinograms from a variety of background adaptation conditions for a C57Bl/6J control group (black), the *Cacna1f* ^G305X^ KO mice (*red*) and the two individual *iZEG:Cacna1f^+^;Pax6::Cre^+^;Cacna1f^G305X^* mice (*green squares* and *triangles*). Background intensities are in cd ∙ m^−2^, and *vertical bars with circles* indicate flash onset. (B) Response amplitudes as a function of background intensities: data points are means for each group, *shaded areas* are 99% CI. (C) Representative examples of four to six superimposed cortical evoked potentials, with *horizontal bars* illustrating the amplitude measurement. (D) *Bar graphs* representing mean ± SD for the two groups, with individual data presented as *green squares* and *green triangles*. The control and *Cacna1f* ^G305X^ KO groups are significantly different (*Wilcoxon signed-ranked test), and the data from two treated individuals have no overlap with either groups. (E) Three superimposed single-sweep recordings of superior colliculus activity, collected through a high-impedance electrode. Vertical bar with circle indicates the stimulus onset; most spiking activity occurs with a latency of 40–50 ms. Calibration as indicated.

Functional integrity of *iZEG:Cacna1f^+^;Pax6::Cre^+^;Cacna1f^G305X^* mice (n = 4 eyes in two mice) was investigated using scotopic (Fig. 4) and photopic (Fig. 5A) electroretinograms, as well as cortical evoked potentials (Fig. 5B) and multiunit recordings at the superior colliculus (the main subcortical thalamic recipient of retinal ganglion cells) (Fig. 5C). These results were contrasted to those from *Cacna1f* ^G305X^ KO mice (n= 12 eyes, six animals) and C57Bl/6J control mice (n=26 eyes, 13 animals, data previously reported^41^).

The scotopic electroretinograms of *Cacna1f* ^G305X^ KO mice (Fig. 4A, middle column) showed the typical pattern of electronegative waveform coupled with subnormal a-wave amplitude (Fig. 4C, top graph). At high flash-intensities, the a-wave was followed by a small positive notch, previously proven to be of photoreceptor origin.^20^ No b-wave could be elicited at any flash-intensities (Fig. 4C, middle graph). By contrast, results from the *iZEG:Cacna1f^+^;Pax6::Cre^+^;Cacna1f^G305X^* mice showed a similarly deficient a-wave, but a well-delineated b-wave of normal implicit time with subnormal amplitude (Fig. 4A, right-most waveforms; C, middle graph), with further indications of post-photoreceptor processing (inner retinal, mostly amacrine cells)^46^ in the form of large-amplitude, well-defined oscillatory potentials (Fig. 4B). The b-/a-wave ratio is another measure of post-photoreceptor processing. In the C57Bl/6J control mice, this ratio reached 1.98 [99%CI: 0.23] at high scotopic intensities where mostly cones are responding, and increased to 4.47 [99%CI: 0.68] at −0.6 log cd ∙ s ∙ m^−2^, where both rods and cones contribute to the response (Fig. 4C, Bottom). These values are compatible with the expectation of more photoreceptor to bipolar cell convergence at lower intensities.^47^ The *iZEG:Cacna1f^+^;Cacna1f^G305X^* group (controls) showed a very low b-/a-wave ratio, because the “b-wave” measured at a fixed implicit time of 59 ms is likely to be driven by the recovery phase of the photoreceptor component (0.13 [CI: 0.09] and 0.36 [CI: 0.18], for the cone and cone-rod intensities, respectively). The two *iZEG:Cacna1f^+^;Pax6::Cre^+^;Cacna1f^G305X^* mice (“experimentally rescued retina”) showed positive ratio of 1.21 and 1.20 at high intensities, with indication of convergence by reaching 3.04 and 2.66, at 1.0 and −0.6 stimulus intensities, respectively. The presence of this convergence suggests some reestablishment of the photoreceptor-bipolar cell synapses.

The photopic electroretinograms were also investigated, and data were recorded immediately after the dark-adapted series, starting with the lowest background intensities. The *Cacna1f* ^G305X^ KO mice (Fig. 5A, middle column) showed an a-wave in the mesopic background range (<45-20 cd/m^2^), where rods are contributing to the signal, whereas the signal recorded at high photopic background intensities was nearly extinguished, suggesting no cone activity. In all those recordings, no b-wave component was observed. In contrast, a distinct b-wave was observed in all C57Bl/6J mice, even at high photopic background intensities. This was also the case in *iZEG:Cacna1f^+^;Pax6::Cre^+^;Cacna1f^G305X^* mice, in which a b-wave was present at all background intensities, although with smaller amplitude than in its C57Bl/6J counterpart (Figs. 5A, B). This again suggests partial rescue of photoreceptor-bipolar synaptic transmission. For analysis of post-retinal signal transmission, visually-driven cortical evoked potentials were collected at V1 through pial-surface electrodes. As expected, no activity could be evoked in the *Cacna1f* ^G305X^ KO mice,^20^ but cortical responses of typical waveform and large amplitude were evoked in the *iZEG:Cacna1f^+^;Pax6::Cre^+^;Cacna1f^G305X^* mice (Figs. 5C, D), confirming the presence of photoreceptor-bipolar synaptic transmission, as well as reliable transmission within the visual pathways up to the primary visual cortex in the latter strain. Multi-unit activity recorded at the level of the superior colliculi (Fig. 5E) further confirmed the activation of visually driven post-retinal pathways. From the recordings collected, the multi-unit activity appeared to be as strong in the treated transgenic animals as in C57Bl/6J mice, with response latencies in the same range.

#### Immunohistochemical Analysis

**Figure 6.**
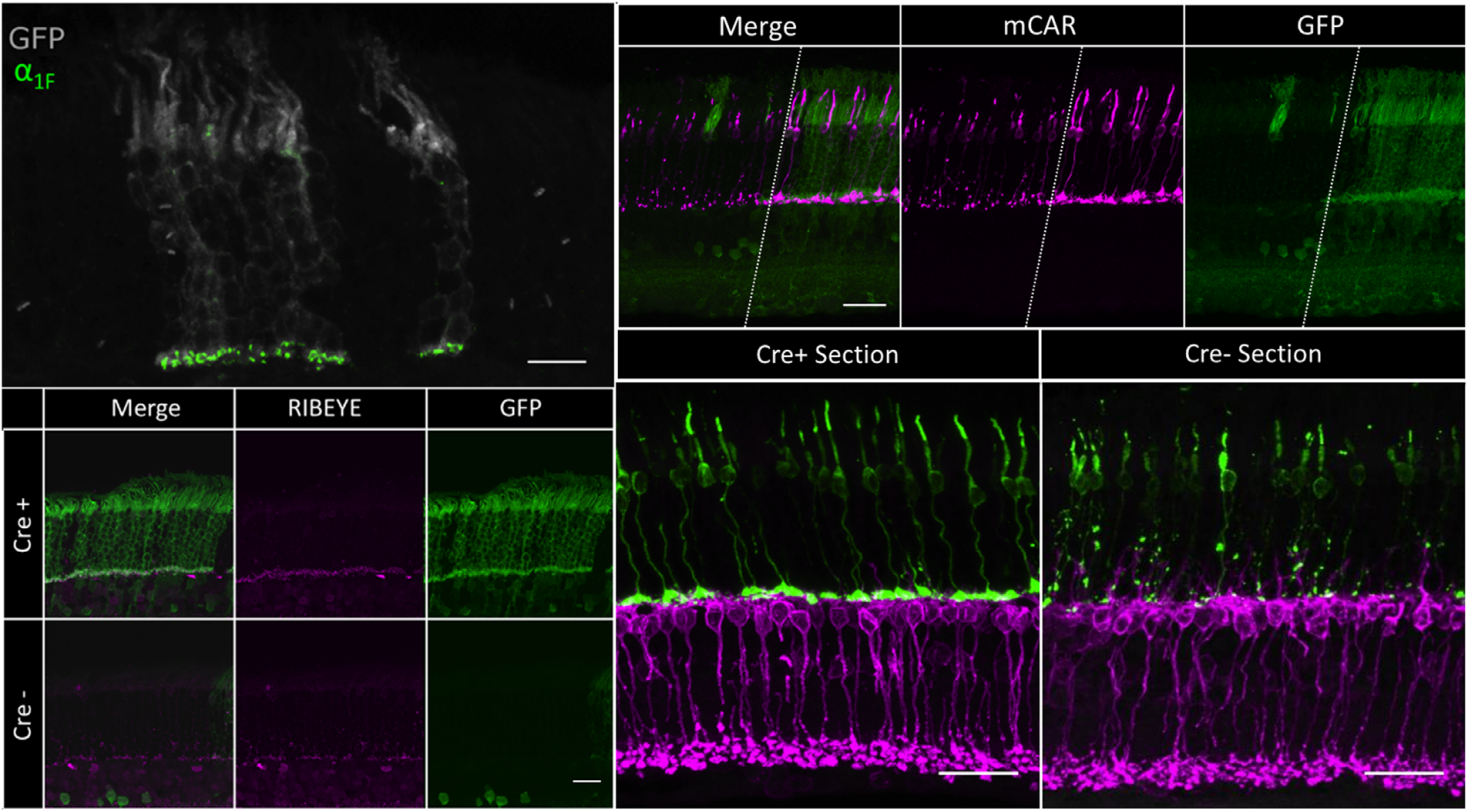
(*Upper Left*) 60× cross-section of a recombined region of an *iZEG:Cacna1f^+^;Pax6*::*Cre^+^;Cacna1f^G305X^* mouse retina, immunolabeled for GFP (*gray*) and α_1F_ (*green*). α_1F_-positive ribbon synapses can be observed in the OPL of GFP-positive columns (where the transgene is expressed and recombined by Cre). *Scale bar*: 10 µm. (*Lower Left*) 60× cross-section of recombined (*top*) and nonrecombined (*bottom*) regions of an *iZEG:Cacna1f^+^;Pax6*::*Cre^+^;Cacna1f^G305X^* mouse retina, immunolabeled for GFP (*green*) and RIBEYE (*magenta*). RIBEYE-positive elongated ribbon synapses can be observed in the OPL of the recombined region, whereas the nonrecombined region contains punctate RIBEYE immunolabeling scattered throughout the OPL and ONL, as previously described in the *Cacna1f*-KO retina.^17^ *Scale bar*: 10 µm. (*Upper Right*) 60x cross-section in a recombined region of an *iZEG:Cacna1f^+^;Pax6*::*Cre^+^;Cacna1f^G305^* mouse retina, immunolabeled for GFP (*green*) and cone arrestin (*magenta*). Cone morphology is preserved in GFP-positive columns (where the transgene is expressed, and recombined by Cre), complete with pedicles, whereas cones in the non-GFP region exhibit signs of CSNB2A-related degeneration. Dotted lines separate recombined and non-recombined regions of the same retinal section. Scale bar = 10 µm (*Bottom Right*) 60x cross-sections of recombined (*left*) and non-recombined (*right*) regions of an *iZEG: Cacna1f^+^; Pax6*: *Cre^+^; Cacna1f^G305X^* mouse retina, immunolabeled for mCAR (*green*) and PKCα (*magenta*). Both cone axons and PKCα-positive bipolar cell dendrites terminate in a monolayer in the OPL in the recombined region, whereas cones exhibit axonal abnormalities and bipolar cells dendrites are sprouting into the ONL in the non-recombined region, as previously reported in the *G305X* mutant retina.^17^^,^^20^^,^^22^ *Scale bar* = 10 µm.

For *iZEG:Cacna1f^+^;Pax6*::*Cre^+^;Cacna1f^G305X^* mice to exhibit any visual function, one would expect to observe signs of rescue of retinal morphology in the peripheral retinal regions expressing both *iZEG:Cacna1f* and *Cre*. Immunohistochemical analyses revealed strong indications of such rescue, including (1) α_1F_ immuno-positive ribbon synapses (Fig. 6, left), (2) preservation of cone morphology (Fig. 6, top right), (3) reduced dendritic sprouting from rod bipolar cells, and (4) mature lamination of cone pedicles (Fig. 6, bottom right). Areas of transgene expression were generally indistinguishable morphologically from wild-type retina labeled for similar markers, although in depth analyses were not performed.^17^

## Discussion

This report describes a novel system for Cre-inducible expression of *Cacna1f* in a *Cacna1f-*KO mouse model of CSNB2A. The *iZEG:Cacna1f* transgene was shown to function as expected, both in vitro and in vivo. Subsequent experiments in *Cacna1f*-KO mice showed that transgenic expression of *Cacna1f* cDNA in the peripheral retina of *iZEG:Cacna1f^+^;Pax6::Cre^+^;Cacna1f^G305X^* mice was able to restore some visual function—and, apparently, to rescue cone, bipolar cell, and photoreceptor synaptic morphology completely—in regions with Cre-mediated transgene activation. Unfortunately, small n-values of transgenic mice precluded statistical analysis of electroretinographic and optokinetic response data. Nonetheless, these preliminary data provide evidence of transgenic rescue that merits further investigation.

Beyond proving the function of the *iZEG:Cacna1f* transgene, the observations here of visual and morphologic rescue in the *iZEG:Cacna1f^+^;Pax6::*C*re^+^;Cacna1f^G305X^* retina address several concerns with respect to expressing *Cacna1f* cDNA. One such concern was the possibility that unregulated expression of *Cacna1f/*α_1F_ (under control of the strong CAG promoter)—causing an excess of α_1F_ protein—might be detrimental to synaptic morphology and thus to visual function. Why did this not happen? We suggest that limited expression of an accessory subunit (β, α_2_δ) necessary for membrane translocation of the channel may be limiting the provision of complete Ca_V_1.4 at the synapse, and thus providing an alternative regulatory mechanism for synaptic calcium regulation.^24^^,^^48^ It should be noted, however, that transgenic *Cacna1f* expression was not experimentally quantified in this work. As such, it is only assumed that the CAG promoter resulted in the expression of supraphysiological levels of α_1F._ Further investigation of this phenomenon is warranted, because it may be relevant to the success of gene therapies for other channelopathies in addition to this one.

Another concern was the use of full-length *Cacna1f* cDNA in the *iZEG:Cacna1f* transgene. While necessary for the procedures involved in transgenesis, compression of the *Cacna1f* gene into cDNA in the vector results in the expression of only a single isoform. With genomic expression, by contrast, alternative splicing of mRNA for voltage-gated calcium channels is extensive, resulting in expression of a myriad of channel isoforms with different kinetics.^49^ More than 20 splice isoforms of *Cacna1f* mRNA have been identified in the human retina, many with markedly diverse biophysical properties.^12^^,^^50^^,^^51^ Expression of only the full-length *Cacna1f* isoform in the *Cacna1f-*KO retina therefore could be suboptimal for enabling the wild-type-like calcium current. Nevertheless, the rescue of some retinal function and morphology observed in these studies suggests that the full-length isoform is sufficient for the formation of functional synapses. This observation is important, not only for this research going forward, but also for potential therapeutic interventions in other channelopathies.

Despite these positive findings, this study also faced some limitations. The mosaic expression of the *iZEG:Cacna1f* transgene in this model is likely the result of nontargeting integration into a genomic locus that is predisposed to epigenetic silencing.^43^^,^^52^ This silencing may also be exacerbated by use of the iZEG system, because the CAG promoter has been shown to be especially susceptible to silencing by DNA methylation.^44^ The minimal visual function observed in the *iZEG:Cacna1f^+^;Pax6::Cre^+^;Cacna1f^G305X^* mice is due, at least in part, to this mosaicism, because only ∼10% to 20% of the total retinal area was found to express the *iZEG*:*Cacna1f* transgene. Additionally, although the use of the *Pax6::Cre* driver for retina-specific recombination is useful for early, constitutive expression, the limitation of its recombination to the peripheral retina likely also limited preservation of normal visual function and OKR. Although suboptimal for measurement of visual function, the mosaic expression in these retinas did conveniently introduce an internal control, which lends further credibility to immunohistochemical analyses of morphologic rescue.

Although the above data provide supportive evidence for the ability of transgenically expressed *Cacna1f* to restore retinal morphology and function in *Cacna1f*^G305X^ mice, the *Pax6::Cre* transgene used in these experiments activates expression during embryonic development.^30^^,^^31^ Thus the question remains: can ectopic expression of *Cacna1f* rescue retinal physiology after birth and/or the typical period of synaptogenesis? This model provides opportunity to answer this question with alternative *Cre* drivers, such as those in which temporally controlled tissue-specific expression can be induced. These include *Cre-ert2*-expressing transgenes. in which recombination is induced by localized treatment with tamoxifen.^53^^,^^54^ If it were bred into the G305X *Cacna1f*-KO background, this inducible system would allow one to establish the developmental window during which transgenic α_1F_ expression can rescue normal retinal structure and function.

Many data suggest that this late-stage rescue is feasible, despite the critical role of α_1F_ in synaptogenesis. Sequential analysis of gene expression of *Cacna1f* suggests that, in mice, expression of the gene begins postnatally.^55^^,^^56^ In support of this, immunohistochemical labeling in wildtype P5 retinae detects only low levels of α_1F_ in the OPL, and wildtype and *Cacna1f* knock-out retinae appear markedly similar with regard to synaptic ribbon markers and bipolar cell processes, up to and including P8.^19^ It has also been established that the maturation of ribbon synapses in mouse photoreceptors is not complete until ∼P14.^57^^,^^58^ These data imply that there is a postnatal time-window, during which expression of wildtype α_1F_ in photoreceptors might be sufficient to support functional synapse formation and vision in *Cacna1f*-deficient mice. Recent evidence even suggests that this window might extend well past the typical period of ribbon-synapse development. A recently published study by Laird et al.^28^ used in vivo electroporation to express inducible *Cacna1f* transiently in the retina of an alternative *Cacna1f*-KO model. Despite limited efficiency in inducing the expression of α_1F_, the authors were able to produce mature photoreceptor synaptic morphology and a suggested improvement in visual function by inducing α_1F_ expression in mice as old as 28 days—well past the typical window of synaptogenesis. The data from our study complement these findings and provide a proof of concept for an alternative model—one that might provide more widespread functional and morphological rescue at later developmental stages, using inducible *Cre* driver lines. Because the majority of CSNB2 cases are diagnosed well after birth, information regarding the potential window of intervention is crucial for the development of therapeutics (such as gene therapy) for this and similar developmental disorders.^1^

The present report, coupled with the research of others into synaptogenesis in models of CSNB2A, has relevance to therapy for CSNB2A—and potentially other common, visually debilitating retinal diseases. Transplantation of photoreceptors is being actively pursued as a means of treating vision-impairing retinal degeneration, but it has recently become apparent that early experiments suggesting functional integration of transplanted photoreceptors were misinterpreted, such that the issue of integration of transplanted photoreceptors into inner-retinal circuitry remains unsolved.^59^ If transgenic expression of *Cacna1f* cDNA can promote the integration of intrinsic photoreceptors into the neural circuitry of *Cacna1f*-KO retinas late in development as suggested by Laird et al.,^28^ then subsequent experiments involving the transplantation of extrinsic wild-type photoreceptors (or precursors) into the subretinal space could be performed in *Cacna1f*-KO retinas. Such experiments might hold the key to understanding how photoreceptor integration can be achieved in retinas in which photoreceptors have been lost because of retinitis pigmentosa, age-related macular degeneration, and many other acquired and inherited retinal degenerative diseases.^12^^,^^60^

## Supplementary Material

Supplement 1
