# Supplementary material for: Transgenic Expression of Cacna1f Rescues Vision and Retinal Morphology in a Mouse Model of Congenital Stationary Night Blindness 2A (CSNB2A)
Source: Transl Vis Sci Technol. 2020 Oct 14;9(11):19. doi: 10.1167/tvst.9.11.19 (PMC7571326; doi:10.1167/tvst.9.11.19)
Supplement: Supplement 1 [file tvst-9-11-19_s001.pdf]

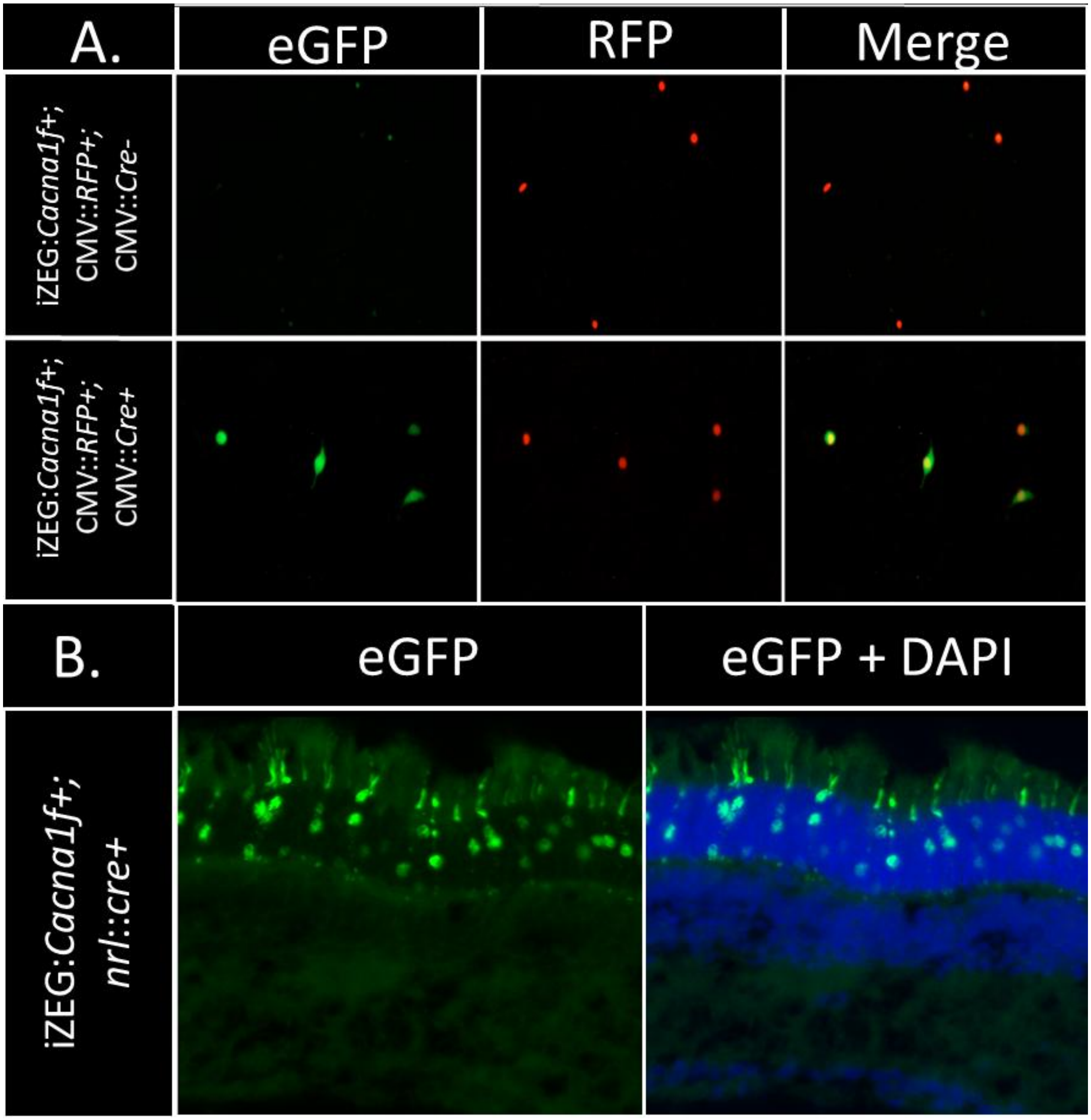

Supplementary Figure 1:

A. *iZEG:Cacna1f* transgene plasmid construct was transfected into HEK 293T cells, with and without the *CMV::Cre* driver plasmid. In the presence of the *Cre* driver, the *iZEG:Cacna1f* vector expresses *eGFP* colocalized with a co-transfected *CMV*-driven nuclear *RFP* reporter plasmid. **B.** Mouse retinas were electroporated *in vivo* with *iZEG:Cacna1f* and *Nrl::Cre* plasmids at P0, and then harvested and imaged at P14.
